# Self-reported psychological distress in childhood and mental health-related hospital attendance among young adults: a 12-year data linkage cohort study from England

**DOI:** 10.1007/s00127-025-02854-y

**Published:** 2025-02-18

**Authors:** Gergő Baranyi, Katie Harron, Nasir Rajah, Emla Fitzsimons

**Affiliations:** 1 Centre for Longitudinal Studies, UCL Institute of Education, University College London, London, UK; 2 Population, Policy & Practice Department, UCL GOS Institute of Child Health, University College London, London, UK

**Keywords:** Adolescence, Cohort study, Administrative data, Mental health, Service use, Treatment gap

## Abstract

**Purpose:**

Investigating the relationship between self-reported mental health and secondary care utilisation can provide evidence on the link between population-level common mental conditions and clinical care; however, cohort studies with linked administrative data are rare. We explored the link between self-reported mental health in adolescence and mental health-related hospital attendance in young adulthood.

**Methods:**

Data from a nationally representative English cohort (Next Steps) were linked to NHS Hospital Episode Statistics. GHQ-12 assessed psychological distress in Next Steps at age 15; participants were followed up until their first mental health-related hospital presentations and outpatient treatments or were censored at the end of the study (age 27). Cox proportionate hazard models with survey weights estimated associations.

**Results:**

Out of 4058 young people, 19% reported high levels of distress at age 15. During the 12-year follow-up, 5.3%, 2.9% and 2.7% of the participants had at least one mental disorder, drug/alcohol misuse and self-harm presentation, respectively, and 4.2% had a mental health treatment in NHS hospitals. Higher GHQ-12 scores were associated with mental disorder presentations (HR = 1.10, 95% CI:1.04–1.16), and mental health treatments (HR = 1.14, 95% CI:1.08–1.20). Associations for hospital treatments were weaker for young people living in deprived areas, or if their main parent had lower education.

**Conclusion:**

Adolescent psychological distress is associated with subsequent hospital attendance in young adulthood, but there might be treatment gaps in service utilisation among more disadvantaged individuals. Detecting youth with mental health difficulties may facilitate early intervention, improve life-course outcomes, and ultimately reduce secondary healthcare use.

**Supplementary Information:**

The online version contains supplementary material available at 10.1007/s00127-025-02854-y.

## Introduction

Mental and substance use disorders are the leading cause of disability among children and young people [1] with the prevalence of depression, anxiety, psychological distress, and suicidal behaviours increasing worldwide [2]. In England, one in five children aged 8 to 16, and over 23% young people aged 17 to 19 had a probable mental disorder in 2023, which is a 60% increase in the younger, and a 130% increase in the older age group since 2017 [3], presenting a serious public policy concern [4]. Mental health among children and young people is particularly important as adult mental disorders root in early life with a peak of incidence in mid-to-late adolescence: the first onset of mental disorders occurs before the age of 18 in half of the population [1]. Moreover, adolescent mental health problems, such as depression, have been shown to predict psychosocial outcomes in adulthood, including educational and employment trajectories [5]. While prevention and early intervention are crucial to minimise the life-course impact, there is a serious gap between treatment need and mental health service use in this age group [6].

Detecting children at risk of socio-emotional problems and mental ill-health– in schools [7], primary care [8], or other children serving settings– has been long advocated by experts [9–11] with considerable support from parents [12, 13]. While early identification and treatment is crucial to provide adequate support and prevention, and enhance recovery [14], and thereby to minimise the impact and reduce associated costs [15], deploying universal screening for mental health problems in childhood is not widely implemented [7]. Understanding whether and how mental health difficulties in adolescence contribute to mental health-related service use in later life can provide further insights into the advantages of population mental health screening scales, identify groups where the treatment gap is larger, and ultimately reduce burden on already stretched services [16]. However, existing research on mental health and service utilisation largely relies on cross-sectional or short-term longitudinal studies [17, 18], and service utilisation is often measured via participants’ (or their guardians’) self-report [19–23], and is prone to recall bias [24]. Data linkages between representative cohort studies and administrative records in the UK [25] and elsewhere [26, 27] could overcome these limitations and benefit mental health guidelines [28], but the question has rarely been investigated [29], and - to our knowledge– not during the transition between adolescence to young adulthood.

The present study addressed these limitations by utilising a nationally representative cohort study of young people linked with administrative data from secondary healthcare. More specifically, we explored *(a)* the association between self-reported psychological distress at age 15 and different mental health-related hospital episodes and treatments between age 15 and 27; and identified *(b)* population groups– across sex, ethnicity, parental education, and area-level deprivation– where the associations differed, providing information about potential treatment gaps.

## Methods

Data were drawn from Next Steps (formerly known as the Longitudinal Study of Young People in England), a nationally representative cohort of people born in England in 1989-90 [30]. The study began in 2004 when participants were 13–14 years old with the sampling of approximately 16,000 young people using a two-stage (i.e., schools, pupils) probability proportional to size sampling procedure, with disproportionate stratification for the most deprived schools and for pupils from major ethnic minority groups [31, 32]. After the initial survey, annual data collections took place until the end of secondary school (i.e. 2010; sweep 7) as the cohort was originally established by the Department of Education; in sweep 4, an ethnic boost was added to the core sample [32]. In 2015/2016 (sweep 8), or around the age of 25, data collection was restarted with successfully recontacting and interviewing 7707 young people from previous sweeps, who were also asked for their consent to link health administrative data (Hospital Episode Statistics [HES]) to the survey. Cohort members were included in the analytical sample if they (a) consented to health data linkage and (b) participated in sweep 2 (April 2005 - September 2005) when they were around 15 years old providing information on self-reported psychological distress and potential confounders. All models included survey weights. We ran main models after excluding participants with missing data; in the sensitivity analyses missing data was imputed. Ethical approval for Next Steps has been gained from the National Health Service (NHS) Research Ethics Committee system; written informed consent was obtained from all participants [31].

### Psychological distress

The 12-item General Health Questionnaire (GHQ-12) was first used to measure psychological distress in Next Steps at sweep 2. GHQ-12 is a valid and reliable screening instrument assessing general psychiatric morbidity [33, 34], with item responses summarized on a scale between 0 and 12 using the GHQ scoring method [33]. Following previous Next Steps analysis the threshold of ≥ 4 was used for psychiatric caseness [35].

### Mental health-related hospital attendance

For consented cohort members, name, sex, date of birth and postcode was used by NHS Digital for data extraction, successfully linking 93.5% of consented participants [36]. For participants not matched with HES records we assumed there were no hospital attendances during the follow-up time, rather than the failure of linkage processes [37]. For this study, we used administrative records from two HES databases: Hospital Episode Statistics Admitted Patient Care (HES APC) and Hospital Episode Statistics Outpatient records (HES OP).

HES APC collects all emergency and planned admissions to NHS hospitals and independent sector providers in England which require a hospital bed [38]. As NHS reimburses 98–99% of all hospital activity in England, the dataset provides universal coverage for the country [38]. Diagnoses received during hospital episodes are coded using the International Statistical Classification of Diseases and Related Health Problems 10th Revision (ICD-10) classification, and from 2007 up to 20 ICD-10 codes can be entered for each hospital episode; the first code is the primary diagnosis, other diagnoses are comorbidities [38]. Combining information on admission method (emergency versus non-emergency [i.e., elective, maternity, other admission]) and all available ICD-10 codes, we computed three outcome groups: *(1)* ‘mental disorders’ were separated into *1a)* emergency, and *1b)* non-emergency; for *(2)* ‘drug/alcohol misuse’ only emergency admissions were considered due to negligible number of other admission types; *(3)* ‘self-harm’-related presentations were all emergency. ICD-10 codes are presented in Table S1, classification was largely based on previous work [39].

HES OP database contains outpatient appointments made in NHS hospitals or activities commissioned by NHS in the independent sector [40]. Only attended appointments were considered. Although HES OP captures ICD-10 diagnostic codes, these are poorly completed [40] and thus not useful for the current investigation. Therefore, we used information on the speciality under which the consultant worked during the period of care and classified a group capturing *4)* ‘mental health treatments’ (see Table S2 for relevant codes).

### Covariates

All covariates were measured in sweep 2. Sex (male, female), age in years at data collection, and ethnicity (white, non-white) were extracted from the main interview conducted with cohort members. Household characteristics included the main parent’s highest qualification (degree [or higher education], lower qualification [GCE A level, GSCE grades A-C, qualifications at level 1 or below, other qualifications], no qualification), main parent’s living status (living with partner, single parent), and the 2004 Income Deprivation Affecting Children Index (IDACI). Main parent was defined as the person most involved in the participant’s education (self-report), which was usually a female (86.4%). We computed groups of area-level deprivation (Q1-Low, Q2, Q3, Q4-High) using quartile thresholds of 2004 IDACI scores based on all English lower super output areas. Finally, health-related covariates included whether the cohort members have ever smoked (yes, no), drunk alcohol (yes, no), and whether they had a disability/long-term illness or health problem (yes, no).

### Statistical analysis

Cox proportional hazard regressions provided estimates of the association between psychological distress and mental health-related hospital attendance using age as timescale. Participants entered the study after sweep 2 data collection ended (01.10.2005) and were followed up until the first mental health-related hospital attendance or censored at the end of the study (31.03.2017), whichever happened earlier. Effect estimates were expressed as Hazard Ratios (HR) with their 95% Confidence Intervals (CIs); Schonefeld residuals did not evidence violation of the proportional hazards assumption. Between sweep 2 and 8 a large number of participants dropped out of the sample (see predictors of non-response in [41]). To account for study design and between sweep attrition– and thus help maintain the cohort’s representativeness– we included survey weights at the time of data linkage consent (i.e., sweep 8) in all statistical analyses unless otherwise indicated.

After presenting weighted descriptive statistics, and weighted cumulative incidence by low and high GHQ-12 scores, three hierarchically adjusted models were conducted. Model 1 adjusted for sex, age, and ethnicity; Model 2 additionally controlled for main parent’s highest qualification, main parent’s living status, and IDACI. Model 3 further included smoking status, drinking status, and disability/long term illness or health problem. We provided effect estimates for the continuous and dichotomised GHQ-12 variables.

Using the fully adjusted models, exploratory analyses investigated whether the association between psychological distress and mental health-related hospital attendance differed by sex, ethnicity, main parent’s highest qualification and IDACI score, by adding interaction terms. Where there was a significant interaction, stratified models were presented to aid interpretation. Finally, we explored the linearity of relationship by adding penalised splines with 2, 3 and 4 degrees of freedom; the best fitting model was determined using the Bayesian Information Criterion (BIC) [42].

Three sensitivity analyses were carried out. First, we imputed missing data for all participants who consented to data linkage using multiple imputation by chained equations based on 7 imputed datasets; number of datasets were determined based on the fraction of missing information. Time-invariant variables were added from sweep 8 (i.e., age, sex, ethnicity), auxiliary variables included household income and housing tenure; and, for each time-variant covariate we included the same variable from nearest available sweeps. Second, for mental disorder as well as drug/alcohol misuse presentations we classified cases where relevant ICD-10 codes were primary diagnoses only, i.e., accounting for the majority of the length of the hospital stay [38], and reran the fully-adjusted models. For self-harm, all diagnostic codes were comorbidities, so it was not included in this sensitivity analysis. Last, we reran main models after excluding participants from the sample with any mental health-related hospital attendance prior to the start of the follow-up (01.10.2005).

Analyses were carried out using the *survival*, *survey*, *jskm* and *mice* packages in R version 4.3.0.

**Fig. 1:**
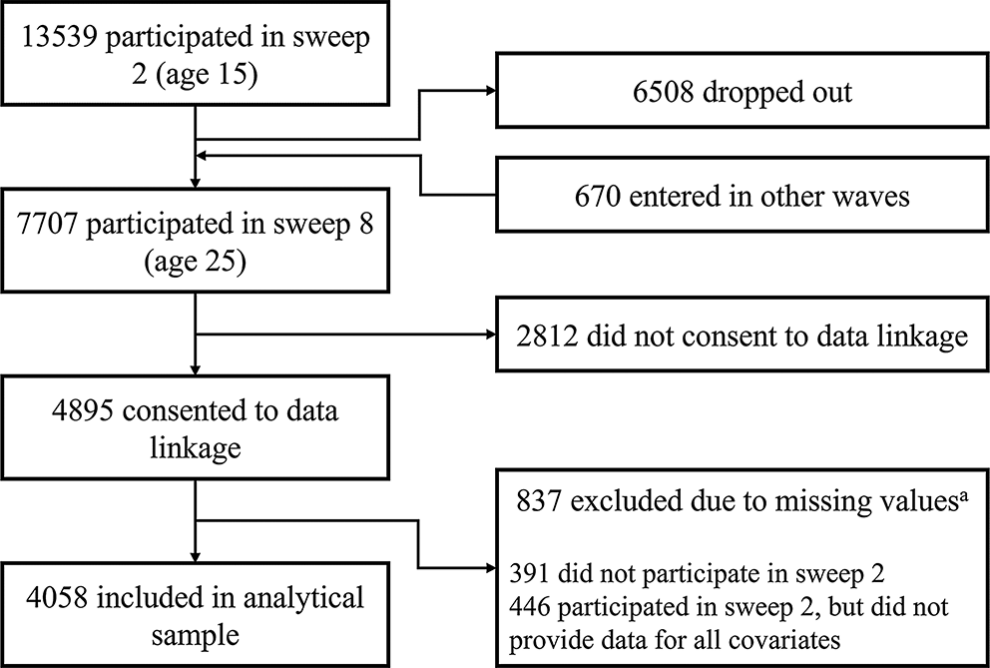
Flowchart depicting sample selection. ^a^ Excluded participants due to missing values were included in the sensitivity analysis using multiple imputations

## Results

Out of 7707 participants present in sweep 8, 4895 consented to health administrative data linkage, of which 4058 young people were included in the analytical sample; 47% of those excluded from the analysis did not participate in sweep 2 where covariates were measured (Fig. 1). Those who consented and not consented health data linkage had some differences in terms of key characteristics: consented participants were more likely male and of white ethnicity, had consumed alcohol before the age of 15, and were more likely to have a low GHQ-12 score (Table 1). At sweep 2, cohort members in the analytical sample were on average 15.3 years old, 54.0% of them were male, 89.5% white, and 18.9% had a GHQ-12 scores ≥ 4. The maximum follow-up time was 137 months, when participants were on average 27.1 years old. During this period 5.3% of young people had at least one hospital presentation related to mental disorders, while 2.9% and 2.7% of the sample presented for drug/alcohol misuse and self-harm, respectively; outpatient records showed that 4.2% of the sample had a mental health treatment (Table 1). Note, the hospital attendances are not mutually exclusive, and there are significant overlaps between diagnostic groups, suggesting comorbidities between mental health-related conditions (Table S3). The median follow-up time until first hospital attendance ranges between 4.7 (mental health treatment) and 8.8 years (non-emergency mental disorders presentation) (Table S4).

**Table 1:** Descriptive statistics for covariates collected at age 15 and mental health-related hospital attendances from age 15 to 27 years, weighted samples

Variable	Participated in sweep 8 (at age 25) *N* = 7707	Consented to linkage in sweep 8^a^		Analytical sample *N* = 4058
		*N* = 4895	*p*-value^d^	
Sweep Two (age 15)				
Age (years), mean ± SD	15.30 ± 0.31	15.30 ± 0.31	0.919	15.30 ± 0.30
* Missing*	*8.52 (735)*	*7.84 (424)*		*0 (0)*
Sex, % (n)				
Male	45.88 (3090)	48.88 (2106)	< 0.001	54.02 (1942)
Female	44.53 (3863)	42.22 (2345)		45.98 (2116)
* Missing*	*9.59 (754)*	*8.90 (444)*		*0 (0)*
Ethnicity, % (n)				
White	77.70 (4895)	80.98 (3382)	< 0.001	89.53 (3127)
Non-white	12.67 (2051)	10.11 (1068)		10.47 (931)
* Missing*	*9.63 (761)*	*8.91 (445)*		*0 (0)*
Main parent’s living status, % (n)				
Single parent	28.30 (1587)	29.17 (1053)	0.188	30.25 (936)
Living with partner	62.75 (5344)	62.74 (3399)		69.75 (3122)
* Missing*	*8.95 (776)*	*8.09 (443)*		*0 (0)*
Main parent’s highest qualification, % (n)				
Degree or higher education	22.27 (1954)	22.93 (1310)	0.027	25.05 (1201)
Lower qualifications	51.39 (3625)	52.24 (2382)		56.70 (2161)
No qualifications	18.17 (1419)	17.32 (794)		18.25 (696)
* Missing*	*8.16 (709)*	*7.50 (409)*		*0 (0)*
IDACI, % (n)				
Q1- Low	22.65 (1781)	23.34 (1196)	0.227	26.06 (1114)
Q2	21.24 (1592)	21.25 (1059)		23.26 (964)
Q3	21.25 (1524)	21.75 (988)		23.69 (892)
Q4 - High	27.06 (2133)	26.51 (1260)		26.99 (1088)
* Missing*	*7.80 (677)*	*7.15 (392)*		*0 (0)*
Ever smoked, % (n)				
Yes	19.16 (1122)	19.76 (745)	0.298	21.70 (688)
No	67.77 (5597)	68.02 (3567)		78.30 (3370)
* Missing*	*13.07 (988)*	*12.22 (583)*		*0 (0)*
Ever drunk alcohol, % (n)				
Yes	58.46 (3936)	61.13 (2697)	< 0.001	69.45 (2549)
No	28.53 (2777)	26.79 (1620)		30.55 (1509)
* Missing*	*13.01 (994)*	*12.08 (578)*		*0 (0)*
Disability/long term illness or health problem, % (n)				
Yes	14.42 (900)	14.50 (589)	0.919	15.44 (529)
No	77.23 (6081)	77.90 (3887)		84.56 (3529)
* Missing*	*8.35 (726)*	*7.60 (419)*		*0 (0)*
GHQ-12, % (n)				
0–3	69.67 (5393)	70.87 (3489)	0.007	81.10 (3286)
4–12	16.67 (1300)	16.46 (814)		18.90 (772)
* Missing*	*13.66 (1014)*	*12.67 (592)*		*0 (0)*
GHQ-12, mean ± SD	1.78 ± 2.59	1.75 ± 2.56	0.170	1.73 ± 2.54
* Missing*	*13.66 (1014)*	*12.67 (592)*		*0 (0)*
Mental health-related hospital attendances from age 15 to 27 years				
1. Mental Disorder presentation^b^, % (n)		5.90 (237)	NA	5.32 (175)
1a. Emergency, % (n)		3.66 (144)	NA	3.53 (115)
1b. Non-emergency, % (n)		2.98 (124)	NA	2.57 (85)
2. Drug/Alcohol Misuse presentation (emergency)^b^, % (n)		2.99 (101)	NA	2.86 (80)
3. Self-Harm presentation (emergency)^b^, % (n)		2.92 (113)	NA	2.74 (85)
4. Mental Health treatment^c^, % (n)		4.87 (191)	NA	4.24 (145)

Percentages, means and SDs are weighted, n-s are unweighted. Abbreviations: GHQ-12 = General Health Questionnaire 12-item version; IDACI = Income Deprivation Affecting Children Index; MP = Main Parent; SD = Standard Deviation

^a^ Consented sample was included in sensitivity analysis with multiple imputation for missing data

^b^ Hospital Episode Statistics Admitted Patient Care dataset (see code list in Table S1)

^c^ Hospital Episode Statistics Outpatient Records dataset (see code list in Table S2)

^d^ p-value indicates the difference between consented and not-consented participants

**Fig. 2:**
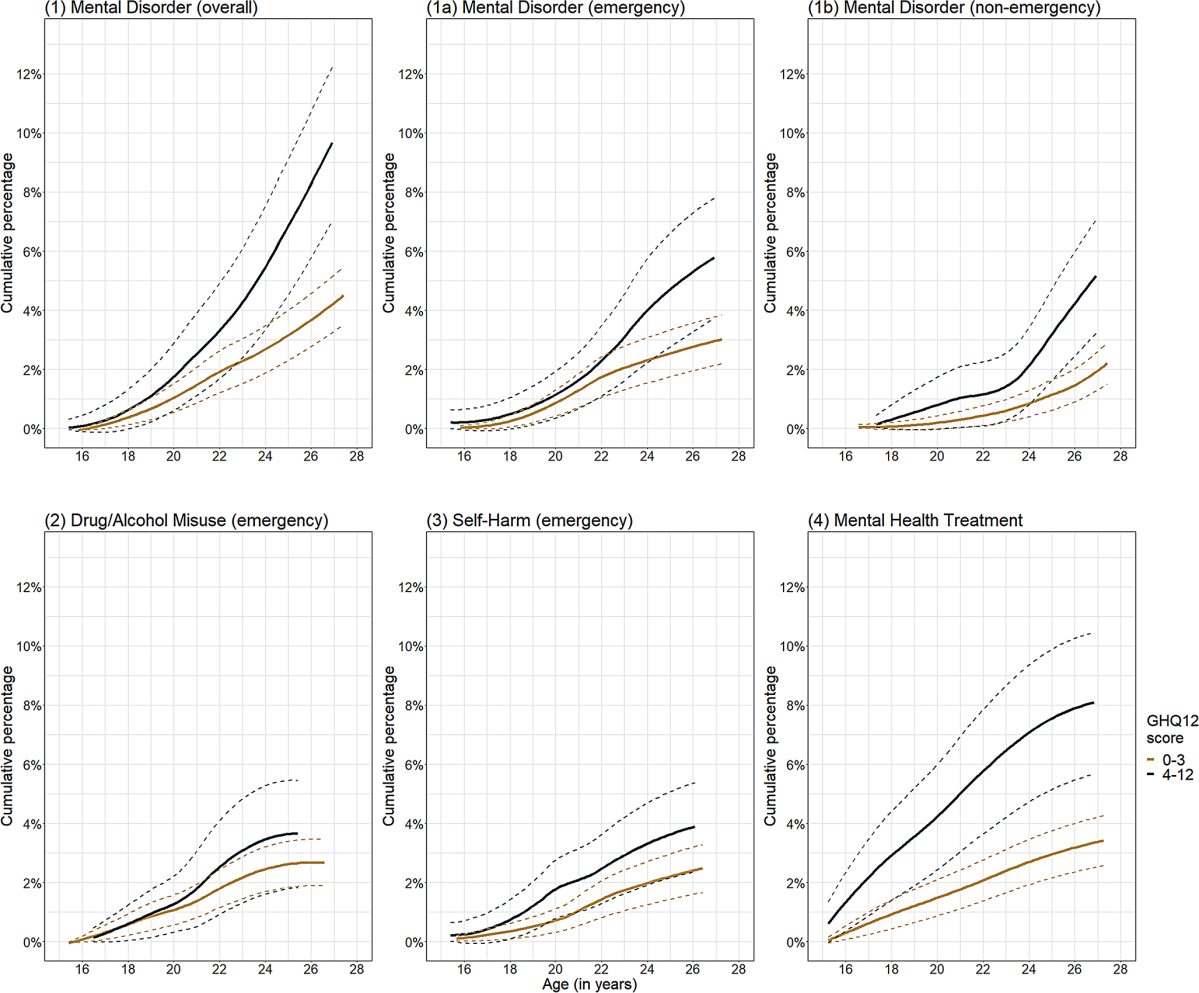
Weighted cumulative incidence of mental health-related hospital attendance from age 15 to 27 years by low (0–3) and high (4–12) GHQ-12 scores; smoothed curves are presented

Figure 2 shows weighted cumulative incidence of mental health-related hospital attendance by low versus high GHQ-12 scores in childhood: for mental disorder presentations (overall) and mental health treatments the difference was more than double (4.3% versus 9.7%, and 3.3% versus 8.1%, respectively). The weighted percentage of low versus high GHQ-12 scores in ethnic and IDACI groups were similar, while there were differences between males and females, and by main parent’s highest qualification (Figure S1).

After adjusting for sex, age, and ethnic groups (i.e., Model 1), GHQ-12 score at age 15 was associated with higher hazard of mental disorder (overall, emergency, non-emergency) and self-harm presentations, as well as mental health treatments (Table 2); further adjustment for socioeconomic factors did not change the findings (i.e., Model 2). In the fully adjusted models, including health-related variables (i.e., Model 3), 1-increase in GHQ-12 score in adolescence was associated with a HR = 1.10 (95% CI: 1.04–1.16) for mental disorder presentations (emergency: HR = 1.08 [95% CI: 1.01–1.15]; non-emergency: HR = 1.12 [95% CI: 1.04–1.20]) and a HR = 1.14 (95% CI: 1.08–1.20) for mental health treatment (Table 2). *Post-hoc*, we estimated the association for mood (ICD-10 codes: F30-F39) and anxiety disorder-related presentations (ICD-10 codes: F40-F49); the two largest groups within mental disorders, allowing separate analyses with still sufficient power. The hazards were identical for mood (HR = 1.11 [95% CI: 1.04–1.18]; number of cases = 120) and anxiety disorder presentations (HR = 1.11 [95% CI: 1.03–1.19]; number of cases = 69). In Model 3, GHQ-12 was not associated with drug/alcohol misuse and self-harm presentations. In comparison to low GHQ-12 scores, high scores approximately doubled the hazard of mental disorder presentations (overall: HR = 1.86 [95% CI: 1.26–2.75]; non-emergency: HR = 2.22 [95% CI: 1.70–3.89]) and mental health treatments (HR = 2.23 [95% CI:1.47–3.38]) in Model 3 (Table S5).

**Table 2:** Continuously measured GHQ-12 scores at age 15 and mental health-related hospital attendance from age 15 to 27 years

	Model 1			Model 2			Model 3		
	HR	95% CI	*p*	HR	95% CI	*p*	HR	95% CI	*p*
1. Mental Disorder (overall)	1.11	1.06–1.17	< 0.001	1.12	1.06–1.17	< 0.001	1.10	1.04–1.16	< 0.001
a. Mental Disorder (emergency)	1.11	1.04–1.17	0.001	1.11	1.04–1.18	0.001	1.08	1.01–1.15	0.02
b. Mental Disorder (non-emergency)	1.11	1.04–1.19	0.002	1.12	1.04–1.20	0.001	1.12	1.04–1.20	0.002
2. Drug/Alcohol Misuse (emergency)	1.06	0.98–1.15	0.17	1.06	0.98–1.15	0.17	1.04	0.95–1.13	0.39
3. Self-Harm (emergency)	1.09	1.01–1.16	0.02	1.09	1.02–1.17	0.01	1.06	0.99–1.15	0.11
4. Mental Health Treatment	1.16	1.10–1.22	< 0.001	1.15	1.09–1.22	< 0.001	1.14	1.08–1.20	< 0.001

Cox proportionate hazard regressions were fitted using survey weights; Hazard Ratios (HR) and their 95% confidence intervals (CI) are presented. Sample size was *N* = 4058

Model1: adjusted for sex, age, and ethnicity

Model2: Model 1 + main parent’s living status, main parent’s highest qualification, and Income Deprivation Affecting Children Index

Model3: Model 2 + ever smoked, ever drunk alcohol, and having disability/long term illness or health problem

**Fig. 3:**
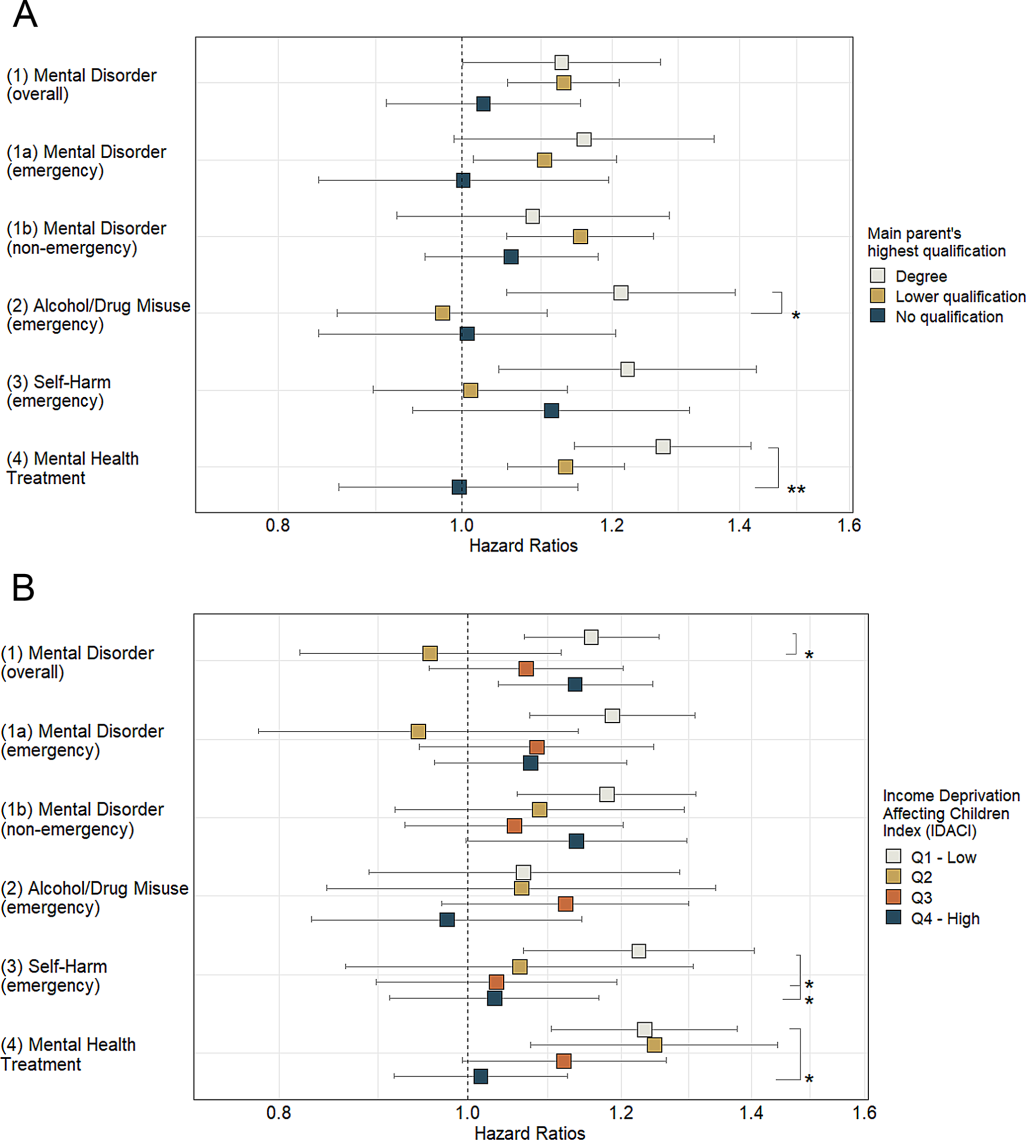
Associations between continuously measured GHQ-12 scores at age 15 and mental health-related hospital attendance from age 15 to 27 years by (**A**) main parent’s highest qualification and by (**B**) Income Deprivation Affecting Children Index. Effect estimates are expressed in Hazard Ratios and their 95% Confidence Intervals; stars indicate a significant interaction between respective groups at *p* < 0.05 (*) and *p* < 0.01 (**) level. Models were adjusted for sex, age, ethnicity, main parent’s living status, main parent’s highest qualification (B only), Income Deprivation Affecting Children Index (A only), ever smoked, ever drunk alcohol, and having disability/long term illness or health problem. Sample sizes were 1201, 2161 and 696 for ‘degree’, ‘lower qualification’ and ‘no qualification’ groups, and 1114, 964, 892 and 1088 for ‘Q1– Low’, ‘Q2’, ‘Q3’, and ‘Q4- High’ deprivation groups, respectively

Hospital attendance differed across sex, ethnicity, parental education and area-level income deprivation: females (apart of Drug/Alcohol Misuse), young adults from white ethnic, and lower educated parental backgrounds, as well as from deprived areas, had more mental health-related hospital presentations and treatments (Figure S2). Sex or ethnic groups did not modify the association between self-reported mental health and hospital attendance (Table S6). In comparison to young people with degree-holding main parents, for cohort members where main parent had a lower or no qualification the hazard of drug/alcohol misuse presentations and mental health treatment were lower and non-significant (Fig. 3A). Similarly, in comparison to young people from advantaged neighbourhoods (Q1), those from deprived areas had a lower hazard of overall mental disorder (Q2), and self-harm presentations (Q3, Q4), as well as mental health treatments (Q4) (Fig. 3B). These associations were particularly striking as there was a clear social gradient for different types of hospital attendance (Figure S2). Effect modification by parental education and area disadvantage remained significant for mental health treatment even after adjusting for false discovery rate (Table S6). Finally, we did not find evidence for non-linear relationship between GHQ-12 and mental health-related hospital attendance (Table S7, Figure S3).

In sensitivity analyses, the above associations only marginally changed. Multiple imputation addressing missing data led to almost identical estimates in the fully adjusted model (Table S8). Using ICD-10 codes only from the primary diagnostic column of HES APC, reduced substantially the number of events, but it did not markedly change the findings for mental disorder (HR = 1.13 [95 CI: 1.04–1.22]; number of cases = 28) and drug/alcohol misuse presentations (HR = 0.99 [95 CI: 0.79–1.26]; number of cases = 19). Finally, after excluding 48 individuals with prior mental health-related hospital attendance led to very marginal changes (Table S9).

## Discussion

Using a large and nationally representative cohort with almost 12 years of follow-up we demonstrated that reporting psychological distress in adolescence was associated with increased risk of secondary mental health care use in England. Associations were consistent for mental health treatments and mental disorders-related presentations, especially in non-emergency settings. Moreover, we found that youth from families where the main parent had lower educational qualifications and those living in more deprived neighbourhoods had a lower likelihood of having mental health-related hospital treatments despite reporting psychological distress in adolescence.

In line with our findings, previous evidence demonstrated that childhood and adolescence mental health difficulties associate with mental health-related service use, while there is still a large gap between treatment need and service utilisation [18–22, 29]. Self-reported data on mental health-related residential service use (including hospitals stays) suggests that internalising behaviour in preadolescence is associated with higher probability of service use especially during transition to high school [22]. Depression in adolescence has been also associated with higher risk of self-reported specialist mental health service use and service use for emotional problems [20]. Similarly, psychiatric diagnoses were also associated with mental health service utilisation [21, 23]. Studies with linked health administrative data show similar findings. Poor self-reported mental health is strongly linked with redeeming prescriptions of antidepressants among youth [18]. More closely to our study, a recent Australian investigation showed mental health-related attendance by self-reported symptom trajectories between age 4 and 14, and suggested that even among children with high levels of problems, less than 40% had an episode of care meeting minimally adequate treatments defined by guidelines [29]. Our study extended this research by confirming the longitudinal associations from adolescence to young adulthood, with slightly stronger associations for non-emergency attendance.

Identifying children at risk of mental ill-health or with undiagnosed mental disorders, can lead to early treatments [9, 11], provide significant economic benefits [15] and lower the burden on already stretched services [15, 16]. Although GHQ-12 is a checklist design to detect depressive and anxiety symptoms [43], and post-hoc analyses confirmed associations with anxiety and mood disorders specifically, it is important to stress that typically those with more severe mental health problems are presented or treated in secondary care settings. Primary care is usually the first point of contact for individuals with mental health care needs: in 2019, 26.6% of total GP consultations in England were either for mental health, or for a combination of mental and physical health problems with higher proportions among younger population groups [44]. In the same year, only 4.9% of the English population were in contact with secondary mental health services and only fraction of these (3.8%, or 0.2% of the total population) required an overnight stay [45]. Findings from our study on young adults showed comparable proportions; despite 19% of the sample having high levels of psychological distress at age 15, only 4% of the overall sample accessed hospital-based secondary mental health treatment in the following 12 years (or 8.1% of those with high GHQ-12 scores). Overnight stay due to mental disorders as the primary diagnosis were very rare in the sample (< 1%).

Although females and white ethnic participants were more likely to have a mental health-treated secondary care episode during the follow-up, this did not change the association between self-reported psychological distress and hospital attendance. We found effect modification by parental education and area-deprivation, where psychological distress was only associated with hospital-based mental health treatments among young people from less disadvantaged backgrounds, suggesting a potential treatment gap. Seeking and accessing professional help is influenced by a wide range of factors, including knowledge, stigma, and perception of help-seeking and confidentiality, and also by systemic and structural factors such as costs, transportation difficulties and availability of help [46]. Young people in deprived areas are less likely to receive support or need to wait longer for specialist services [47]. A similar relationship was found for hospital presentations, especially for self-harm; however, these did not survive false discovery rate correction and thus need to be confirmed in future studies. Most young people who self-harm do not seek professional services [48], leaving those from disadvantaged areas less likely to interact with healthcare; potential barriers are similar to other mental health problems [48]. Alternatively, the lack of association in the disadvantaged groups can also indicate mental health stigma affecting the reporting of distress symptoms among adolescents, as attitudes towards mental illness are most stigmatising in disadvantaged areas [49]. Finally, in contrary to US findings [23], we did not find indication of a treatment gap among non-white participants after controlling for other confounders, including various measures of socioeconomic status; however, due to the small sample sizes of specific ethnic groups, we were not able to disaggregate non-white groups, and it is plausible that more detailed analyses would have led to different findings. Moreover, data from community mental health care settings, or involuntary psychiatric care [50], could show different associations.

### Strengths and limitations

This study benefited from a large and representative cohort study linked with hospital administrative records, taking advantage of both types of data. Next Steps contains rich information about key sociodemographic and health variables [31] important to consider in mental health research; NHS HES captures 98–99% of hospital episodes occurring in England, and is thus likely not affected by selection bias [38]. The longitudinal design with close to 12 years of follow-up between adolescence and young adulthood is a unique feature of our study. However, there are limitations. First, 36% of the sample at sweep 8 did not consent to health data linkage. Although we applied survey weights in all analyses, there were differences between consented and not-consented participants in their baseline characteristics (e.g., sex, ethnicity). Help seeking behaviour or the severity of mental health conditions can differ across these characteristics, which might have affected our findings. Second, HES does not contain all mental health-related secondary care service use data from the NHS system, thus we likely underestimated service use in our sample. As opposed to the 12-year prevalence of 4.2% in our sample, annual data from Mental Health Services Data Set (MHSDS)– capturing contact with Mental Health Services– suggest that approximately 5% of the 20–29 years old population in England was in contact with secondary mental health, learning disabilities and autism services in 2018/2019 [45]. As MHSDS was first introduced in 2016 almost 11 years later than our follow-up started, we could not include this database in our analyses. More research is needed to systematically assess strength of associations between self-reported mental health and primary, secondary and community care service use data. Third, access to secondary health care might be affected by geographic accessibility to hospitals and other factors, variables not available in this study. Fourth, we were only able to look at white/non-white differences, future studies with larger samples should explore associations by different ethnic groups. Fifth, 6.5% of the consented sample was not matched, which we included as non-attendance in our analyses [37]. Although there is underascertainment due to linkage error, linkage success is unlikely to depend on mental health status, thus biasing the reported associations. Last, as mental health hospital presentations were mainly relying on comorbidities entered into the HES APC database, systematic differences in how health professionals record hospital episodes might have affected our findings.

## Conclusion

By using an easily implementable mental health screening scale we demonstrated that self-reported psychological distress increases the risk of future mental health-related hospital attendance; however, associations may differ by social background. Future studies should explore a larger range of mental health service use databases, including community-based services and data from primary care (e.g., psychotropic prescriptions). Differentiation between primary and additional diagnostic codes, as well as comorbidities between conditions, requires further attention and larger datasets. Investigating the transition between child and adult services during young adulthood as well as their childhood correlates can benefit from linked cohort and administrative data and can contribute to developing mental health guidelines.

## Electronic supplementary material

Below is the link to the electronic supplementary material.

Supplementary Material 1

## Data Availability

The Centre for Longitudinal Studies, University College London owns the copyright for the Next Steps (formerly known as the Longitudinal Study of Young People in England) data used in this study. The Next Steps data are held/curated by the UK Data Service (https://beta.ukdataservice.ac.uk/datacatalogue/studies/study?id=5545; https://beta.ukdataservice.ac.uk/datacatalogue/studies/study?id=8681). Anyone wishing to use the Next Steps data must register and submit a data request to the UK Data Service at http://ukdataservice.ac.uk/. The Linked Health Administrative Datasets (Hospital Episode Statistics) (SN 8681) is a secure access dataset requiring a Secure Access User Agreement (https://ukdataservice.ac.uk/help/secure-lab/secure-access-user-agreement/); projects including secure access data have to be completed within the UKDS SecureLab. Additional terms and conditions of access are outlined here: https://www.ukdataservice.ac.uk/get-data/how-to-access/conditions. This data can be also access via UKLLC (https://ukllc.ac.uk/apply).
