# Supplementary material for: Self-reported psychological distress in childhood and mental health-related hospital attendance among young adults: a 12-year data linkage cohort study from England
Source: Soc Psychiatry Psychiatr Epidemiol. 2025 Feb 18;60(7):1659–70. doi: 10.1007/s00127-025-02854-y (PMC12238145; doi:10.1007/s00127-025-02854-y)
Supplement: Supplementary file 1 — Supplementary Material 1 [file 127_2025_2854_MOESM1_ESM.docx]

**Supporting Information**

**Table S1**: ICD-10 diagnostic code list for hospital presentations.

**Table S2**: Treatment specialities under which the consultant worked.

**Table S3:** Overlaps between different diagnostic groups among all hospital episodes and in the final sample.

**Table S4:** Weighted median follow-up time in months until first mental health-related hospital attendances.

**Figure S1**: Weighted percentage of low (0-3) versus high (4-12) GHQ-12 scores by sex, ethnic groups, main parent’s highest qualification and by Income Deprivation Affecting Children Index.

**Table S5**: High GHQ-12 scores at age 15 and mental health-related hospital attendance from age 15 to 27 years.

**Figure S2**: Weighted percentage of mental health-related hospital attendances from age 15 to 27 years by main parent’s highest qualification and by Income Deprivation Affecting Children Index.

**Table S6**: Interaction effects between continuously measured GHQ-12 at age 15 and sex, ethnicity, main parent’s highest qualifications and area deprivation on mental health-related hospital attendance from age 15 to 27 years.

**Table S7**: Model fits indices with penalised splines.

**Figure S3**: Dose-response curves between continuously measured GHQ-12 scores at age 15 and mental health-related hospital attendances from age 15 to 27 years.

**Table S8**: Continuously measured GHQ-12 scores at age 15 and mental health-related hospital attendances from age 15 to 27 years, after multiple imputations.

**Table S9:** Continuously measured GHQ-12 scores at age 15 and mental health-related hospital attendance from age 15 to 27 years, after excluding participants with prior mental health hospital attendance.

**Table S1**: ICD-10 diagnostic code list for hospital presentations.

| **Group** | **ICD-code** | **ICD-10 Description** |
| --- | --- | --- |
| Mental Disorders | F20-F29 | Schizophrenia, schizotypal and delusional disorders |
|  | F30-F39 | Mood [affective] disorders |
|  | F40-F49 | Neurotic, stress-related and somatoform disorders |
|  | F50-F59 | Behavioural syndromes associated with physiological disturbances and physical factors |
|  | F60-F69 | Disorders of adult personality and behaviour |
|  | F90-F98 | Behavioural and emotional disorders with onset usually occurring in childhood and adolescence |
|  | F99 | Unspecified mental disorder |
| Drug/ Alcohol Misuse | F10 | Mental and behavioural disorders due to use of alcohol |
|  | F11 | Mental and behavioural disorders due to use of opioids |
|  | F12 | Mental and behavioural disorders due to use of cannabinoids |
|  | F13 | Mental and behavioural disorders due to use of sedatives or hypnotics |
|  | F14 | Mental and behavioural disorders due to use of cocaine |
|  | F15 | Mental and behavioural disorders due to use of other stimulants, including caffeine |
|  | F16 | Mental and behavioural disorders due to use of hallucinogens |
|  | F18 | Mental and behavioural disorders due to use of volatile solvents |
|  | F19 | Mental and behavioural disorders due to multiple drug use and use of other psychoactive substances |
|  | F55 | Abuse of non-dependence-producing substances |
|  | T51 | Toxic effect of alcohol |
|  | Y15 | Poisoning by exposure of alcohol, undetermined intent |
|  | Y90 | Evidence of alcohol involvement determined by blood alcohol level |
|  | Y91 | Evidence of alcohol involvement determined by level of intoxication |
| Self-Harm | X60-X69 | Intentional self-poisoning (drugs, alcohol, gases, other) |
|  | X70-X84 | Intentional self-harm (hanging, drowning, firearm, explosive material, fire, steam, sharp/blunt object, jumping, crashing motor vehicle, other) |

Classification was largely based on Blackburn et al., 2021. Main differences included: i) ICD-10 codes across both primary and other diagnostic positions were considered; ii) only 3-character length ICD-10 codes were available in our study, thus 4-character codes could not be captured; iii) Drug and Alcohol Misuse did not include F17 (Mental and behavioural disorders due to use of tobacco); iv) a narrower definition of Self-Harm was applied only capturing intentional self-poisoning and self-harm.

**Table S2**: Treatment specialities under which the consultant worked.

| **Group** | **Code** | **Description** |
| --- | --- | --- |
| Mental Health Treatment | 656 | Clinical Psychology |
|  | 659 | Drama Therapy |
|  | 660 | Art Therapy |
|  | 661 | Music Therapy |
|  | 710 | Adult Mental Illness |
|  | 711 | Child And Adolescent Psychiatry |
|  | 712 | Forensic Psychiatry |
|  | 713 | Psychotherapy |
|  | 720 | Eating Disorders |
|  | 721 | Addiction Services |
|  | 722 | Liaison Psychiatry |
|  | 723 | Psychiatric Intensive Care |
|  | 724 | Perinatal Psychiatry |
|  | 725 | Mental Health Recovery And Rehabilitation Service |
|  | 726 | Mental Health Dual Diagnosis Service |

**Table S3:** Overlaps between different diagnostic groups among A) all hospital episodes and B) in the final sample.

1. All hospital episodes (n=12991)

| Hospital presentations |  | Mental Disorders | Drug/Alcohol Misuse | Self-Harm |
| --- | --- | --- | --- | --- |
|  |  | *out of 558* | *out of 182* | *out of 203* |
| Mental Disorders | *out of 558* | 558 |  |  |
| Drug/Alcohol Misuse | *out of 182* | 46 | 182 |  |
| Self-Harm | *out of 203* | 83 | 56 | 203 |

| Hospital presentations |  | Mental Disorders | Drug/Alcohol Misuse | Self-Harm |
| --- | --- | --- | --- | --- |
|  |  | *out of 175* | *out of 80* | *out of 85* |
| Mental Disorders | *out of 175* | 175 |  |  |
| Drug/Alcohol Misuse | *out of 80* | 30 | 80 |  |
| Self-Harm | *out of 85* | 53 | 35 | 85 |

1. Analytical sample (n=4058)

**Table S4:** Weighted median follow-up time in months until first mental health-related hospital attendances.

|  | Total | |  | High GHQ-12 score  (4-12 points) | |
| --- | --- | --- | --- | --- | --- |
|  | Median | 95% CI |  | Median | 95% CI |
| 1. Mental Disorder (overall) | 89 | 73-102 |  | 99 | 80-108 |
| a. Mental Disorder (emergency) | 73 | 66-87 |  | 84 | 78-106 |
| b. Mental Disorder (non-emergency) | 106 | 100-118 |  | 107 | 97-121 |
| 2. Drug/Alcohol Misuse (emergency) | 65 | 55-77 |  | 60 | 42-84 |
| 3. Self-Harm (emergency) | 69 | 59-80 |  | 60 | 43-80 |
| 4. Mental Health Treatment | 56 | 49-71 |  | 53 | 25-71 |

N was 4058 for the total sample, and 772 for the high GHQ-12 subsample.

**Figure S1**: Weighted percentage of low (0-3) versus high (4-12) GHQ-12 scores by sex, ethnic groups, main parent’s highest qualification and by Income Deprivation Affecting Children Index.


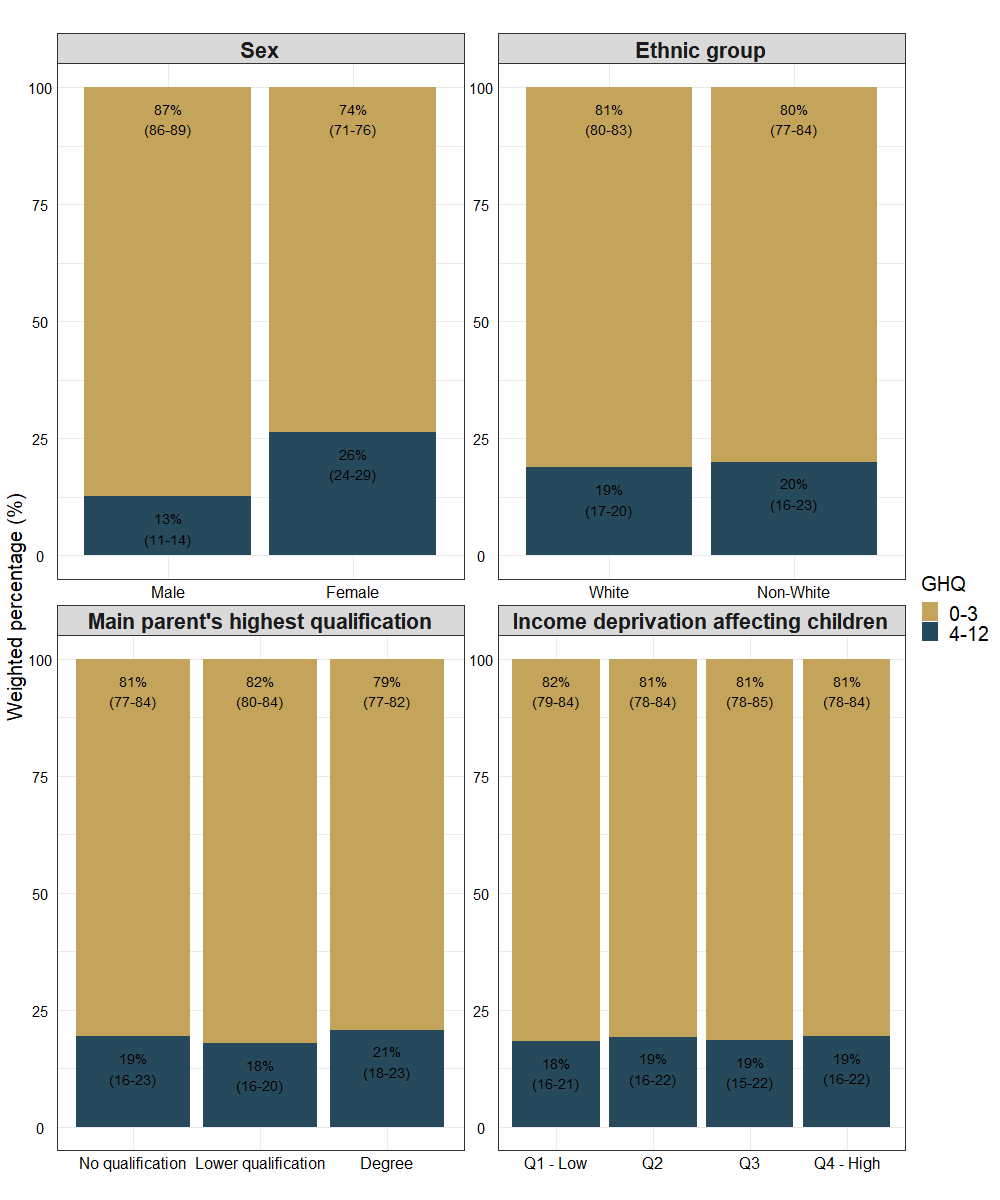


**Table S5:** High GHQ-12 scores at age 15 and mental health-related hospital attendances from age 15 to 27 years.

|  | **Model 1** | | |  | **Model 2** | | |  | **Model 3** | | |
| --- | --- | --- | --- | --- | --- | --- | --- | --- | --- | --- | --- |
|  | HR | 95% CI | p |  | HR | 95% CI | p |  | HR | 95% CI | p |
| 1. Mental Disorder (overall) | 1.98 | 1.37-2.87 | <0.001 |  | 2.00 | 1.37-2.91 | <0.001 |  | 1.86 | 1.26-2.75 | 0.002 |
| a. Mental Disorder (emergency) | 1.72 | 1.08-2.72 | 0.02 |  | 1.72 | 1.08-2.74 | 0.02 |  | 1.50 | 0.93-2.42 | 0.10 |
| b. Mental Disorder (non-emergency) | 2.10 | 1.24-3.57 | 0.006 |  | 2.16 | 1.26-3.68 | 0.005 |  | 2.22 | 1.27-3.89 | 0.005 |
| 2. Drug/Alcohol Misuse (emergency) | 1.59 | 0.86-2.91 | 0.14 |  | 1.55 | 0.85-2.82 | 0.15 |  | 1.40 | 0.74-2.66 | 0.30 |
| 3. Self-Harm (emergency) | 1.49 | 0.90-2.46 | 0.12 |  | 1.49 | 0.90-2.47 | 0.12 |  | 1.32 | 0.77-2.24 | 0.31 |
| 4. Mental Health Treatment | 2.39 | 1.59-3.60 | <0.001 |  | 2.33 | 1.54-3.52 | <0.001 |  | 2.23 | 1.47-3.38 | <0.001 |

Cox proportionate hazard regressions were fitted using survey weights; Hazard Ratios (HR) and their 95% confidence intervals (CI) are presented. High GHQ scores were defined as having 4 to 12 points; low scores (0-3) were used as reference. Sample size was N=4058.

Model1: adjusted for sex, age, and ethnicity.

Model2: Model 1 + main parent’s living status, main parent’s highest qualification, and Income Deprivation Affecting Children Index.

Model3: Model 2 + ever smoked, ever drunk alcohol, and having disability/long term illness or health problem.

**Figure S2**: Weighted percentage of mental health-related hospital attendances from age 15 to 27 years by (A) sex, (B) ethnic groups, (C) main parent’s highest qualification and (D) by Income Deprivation Affecting Children Index.

(A)


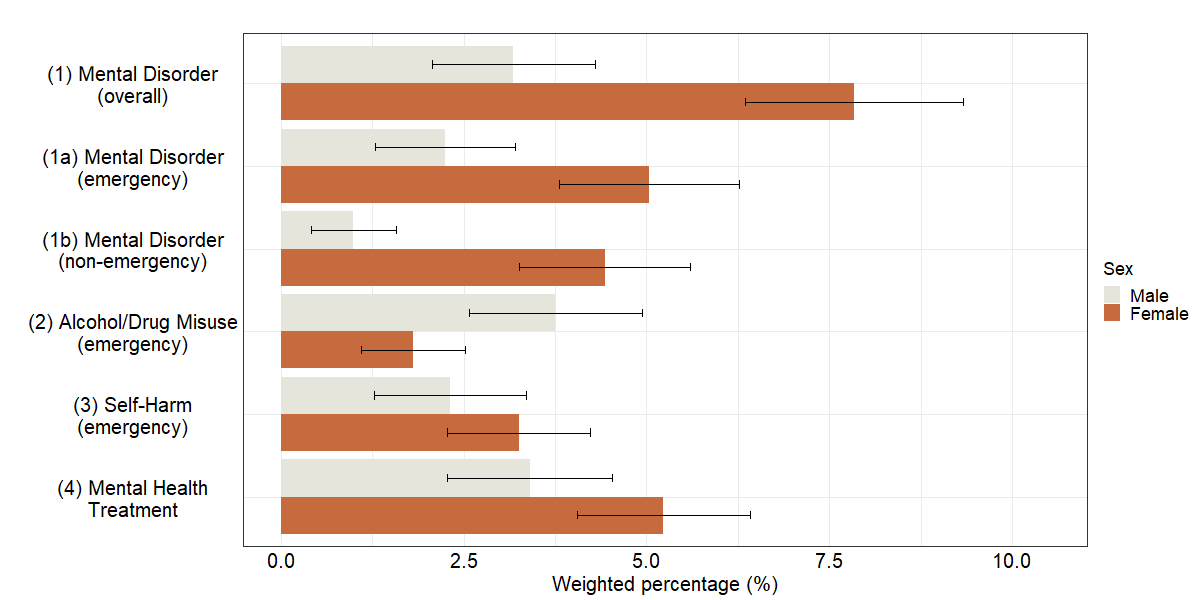


(B)


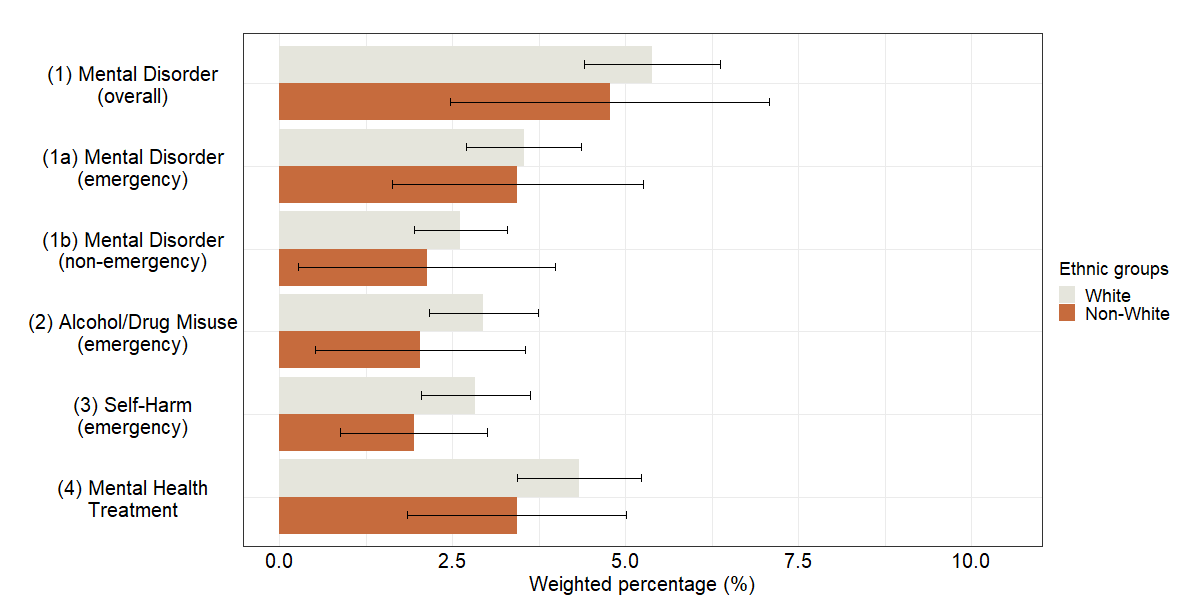


(C)


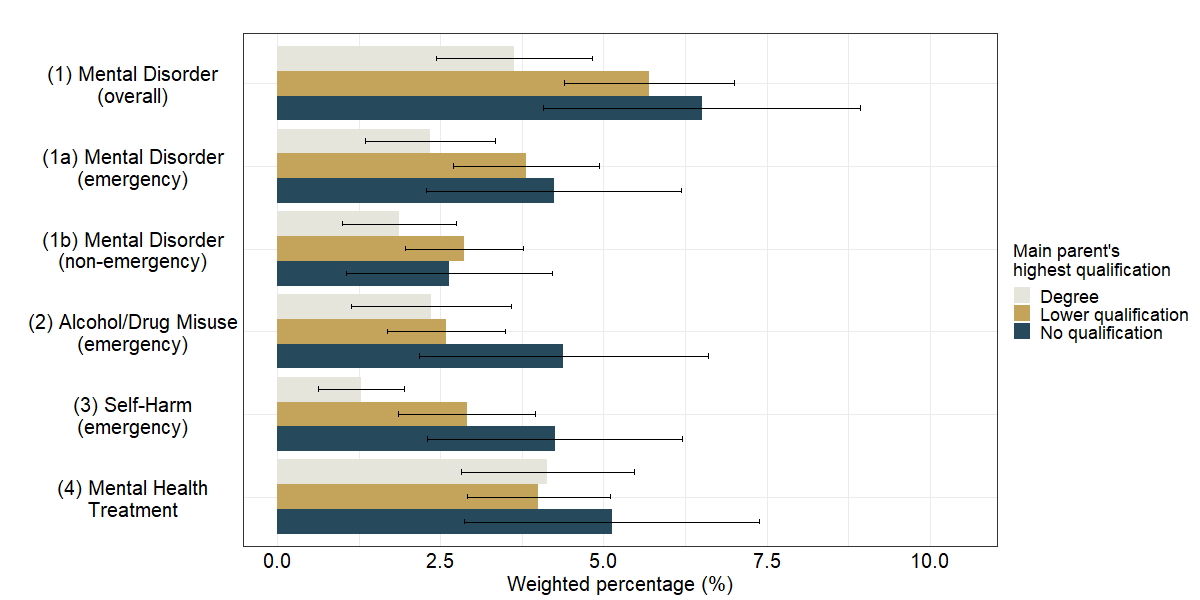


(D)


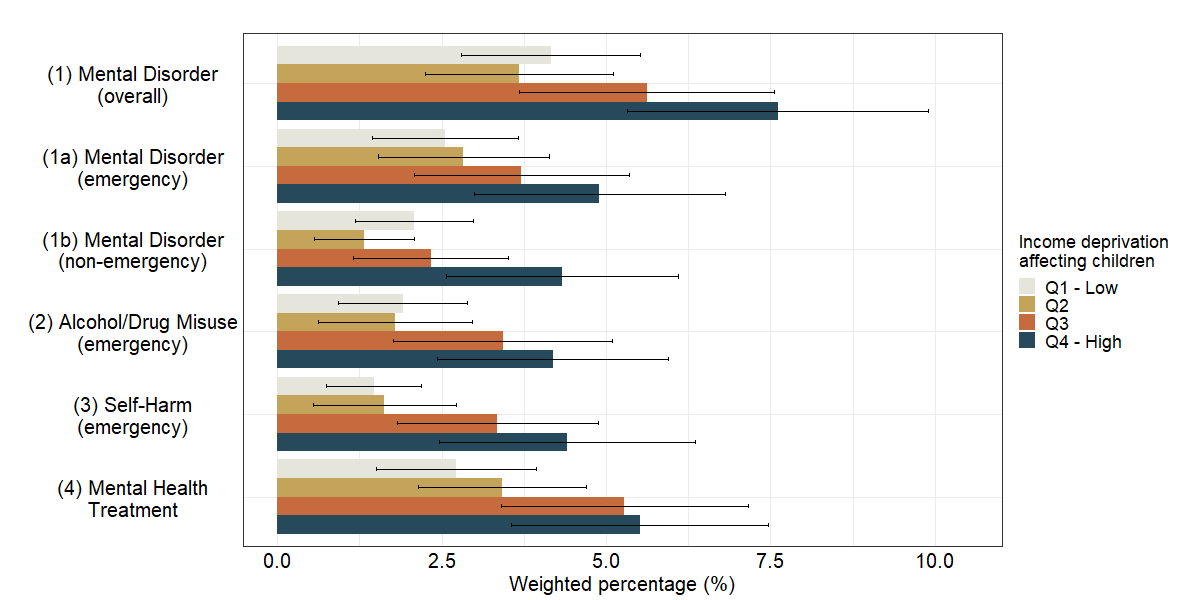


**Table S6:** Interaction effects between continuously measured GHQ-12 scores at age 15 and sex, ethnicity, main parent’s highest qualifications and area deprivation on mental health-related hospital attendances from age 15 to 27 years.

|  | **1. Mental Disorder**  **(overall)** | | |  |  | **1a. Mental Disorder**  **(emergency)** | | |  |  | **1b. Mental Disorder**  **(non-emergency)** | | | |  |
| --- | --- | --- | --- | --- | --- | --- | --- | --- | --- | --- | --- | --- | --- | --- | --- |
|  | HR | 95% CI | p | p_FDR_ |  | HR | 95% CI | p | p_FDR_ |  | HR | 95% CI | p | | p_FDR_ |
| Sex (ref female) | | | | | | | | | | | | | |  | |
| GHQ * Male | 1.00 | 0.88-1.14 | 0.98 | 0.98 |  | 0.98 | 0.85-1.13 | 0.77 | 0.77 |  | 1.10 | 0.88-1.36 | 0.41 | | 0.69 |
| Ethnic groups (ref White) | | | | | | | | | | | | | |  | |
| GHQ * Non-White | 0.95 | 0.78-1.15 | 0.78 | 0.98 |  | 0.96 | 0.77- 1.20 | 0.77 | 0.77 |  | 0.82 | 0.58-1.15 | 0.58 | | 0.69 |
| Main parent’s highest qualification (ref Degree) | | | | | | | | | | | | | |  | |
| GHQ * Other qualifications | 1.01 | 0.90-1.17 | 0.89 | 0.98 |  | 0.98 | 0.84-1.14 | 0.76 | 0.77 |  | 0.99 | 0.85-1.15 | 0.86 | | 0.86 |
| GHQ * No qualifications | 0.91 | 0.78-1.05 | 0.19 | 0.51 |  | 0.86 | 0.70-1.05 | 0.13 | 0.30 |  | 0.87 | 0.71-1.06 | 0.15 | | 0.63 |
| IDACI (ref Q1- Low) | | | | | | | | | | | | | |  | |
| GHQ * Q2 | 0.84 | 0.71-0.99 | 0.049 | 0.34 |  | 0.82 | 0.67-1.01 | 0.06 | 0.30 |  | 0.92 | 0.75-1.13 | 0.43 | | 0.69 |
| GHQ * Q3 | 0.92 | 0.80-1.05 | 0.22 | 0.51 |  | 0.92 | 0.77-1.09 | 0.32 | 0.56 |  | 0.88 | 0.73-1.06 | 0.18 | | 0.63 |
| GHQ * Q4 - High | 0.96 | 0.86-1.07 | 0.45 | 0.79 |  | 0.91 | 0.80-1.03 | 0.12 | 0.30 |  | 0.96 | 0.82-1.12 | 0.59 | | 0.69 |
|  | **2. Alcohol/Drug Misuse**  **(emergency)** | | |  |  | **3. Self-Harm**  **(emergency)** | | |  |  | **4. Mental Health**  **Treatment** | | | |  |
| Sex (ref Female) | | | | | | | | | | | | | |  | |
| GHQ * Male | 1.01 | 0.85-1.20 | 0.93 | 0.96 |  | 0.84 | 0.71-1.00 | 0.05 | 0.09 |  | 1.05 | 0.93-1.19 | 0.45 | | 0.63 |
| Ethnic groups (ref White) | | | | | | | | | | | | | |  | |
| GHQ * Non-White | 0.92 | 0.70-1.21 | 0.70 | 0.96 |  | 0.84 | 0.71-1.00 | 0.71 | 0.71 |  | 1.01 | 0.87-1.18 | 0.87 | | 0.87 |
| Main parent’s highest qualification (ref Degree) | | | | | | | | | | | | | |  | |
| GHQ * Other qualifications | 0.80 | 0.66-0.96 | 0.02 | 0.14 |  | 0.84 | 0.71-1.00 | 0.05 | 0.09 |  | 0.90 | 0.80-1.01 | 0.08 | | 0.29 |
| GHQ * No qualifications | 0.85 | 0.69-1.05 | 0.13 | 0.46 |  | 0.90 | 0.73-1.08 | 0.24 | 0.34 |  | 0.77 | 0.66-0.91 | 0.002 | | 0.01 |
| IDACI (ref Q1- Low) | | | | | | | | | | | | | |  | |
| GHQ * Q2 | 0.99 | 0.75-1.30 | 0.96 | 0.96 |  | 0.89 | 0.71-1.10 | 0.29 | 0.34 |  | 1.01 | 0.88-1.16 | 0.85 | | 0.87 |
| GHQ * Q3 | 0.97 | 0.77-1.23 | 0.79 | 0.96 |  | 0.81 | 0.67-0.98 | 0.03 | 0.09 |  | 0.91 | 0.78-1.07 | 0.26 | | 0.46 |
| GHQ * Q4 - High | 0.91 | 0.72-1.16 | 0.45 | 0.96 |  | 0.84 | 0.71-0.99 | 0.04 | 0.09 |  | 0.84 | 0.73-0.96 | 0.01 | | 0.04 |

Cox proportionate hazard regressions with survey weights were fitted; Hazard Ratios (HR) and their 95% confidence intervals (CI) are presented. Sample size was N=4058. Model parameters are only shown for interaction terms. All models were adjusted for sex, age, ethnicity, main parent’s living status, main parent’s highest qualification, Income Deprivation Affecting Children Index (IDACI), ever smoked, ever drunk alcohol, and having disability/long term illness or health problem. Separate models were conducted for all four effect modifiers.

**Table S7**: Model fits indices with penalised splines.

|  | **Bayesian Information Criterion** | | |
| --- | --- | --- | --- |
|  | 2 degrees of freedom | 3 degrees of freedom | 4 degrees of freedom |
| 1. Mental Disorder (overall) | 2857.37 | 2861.82 | 2866.75 |
| a. Mental Disorder (emergency) | 1909.86 | 1914.72 | 1919.26 |
| b. Mental Disorder (non-emergency) | 1371.01 | 1373.35 | 1376.83 |
| 2. Drug/Alcohol Misuse (emergency) | 1340.24 | 1344.23 | 1348.34 |
| 3. Self-Harm (emergency) | 1409.93 | 1414.27 | 1418.43 |
| 4. Mental Health Treatment | 2375.79 | 2379.62 | 2382.80 |

Sample size was N=4058. Models were adjusted for sex, age, ethnicity, main parent’s living status, main parent’s highest qualification, and Income Deprivation Affecting Children Index, ever smoked, ever drunk alcohol, and having disability/long term illness or health problem (i.e., Model 3). Survey weights were not applied.

**Figure S2**: Weighted percentage of mental health-related hospital attendances from age 15 to 27 years by (A) sex, (B) ethnic groups, (C) main parent’s highest qualification and (D) by Income Deprivation Affecting Children Index.

(A)


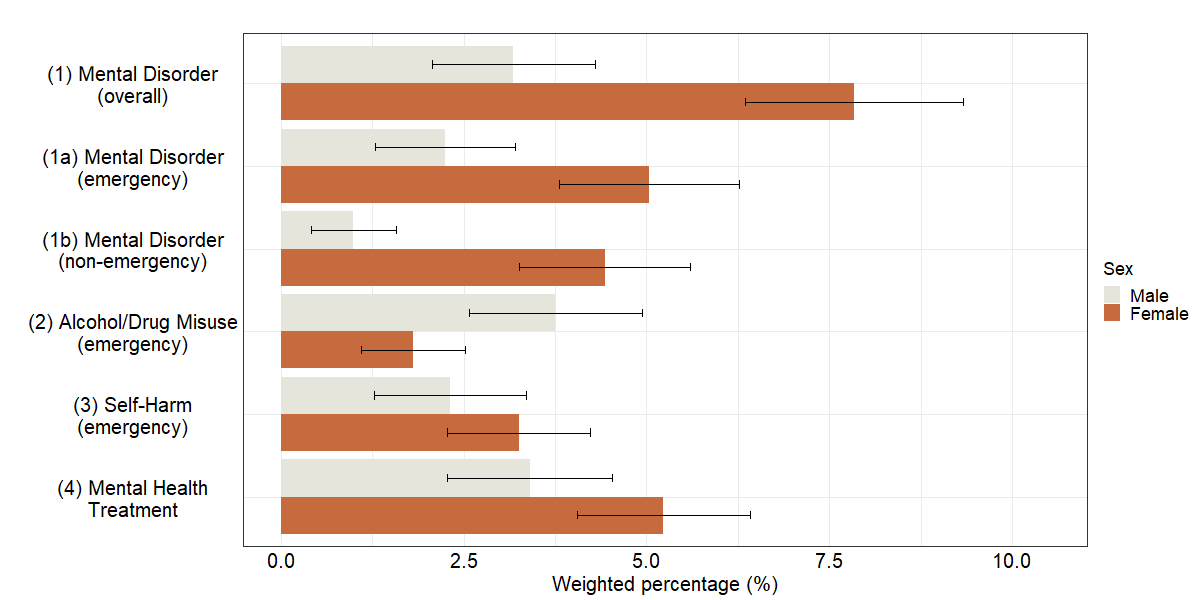


(B)


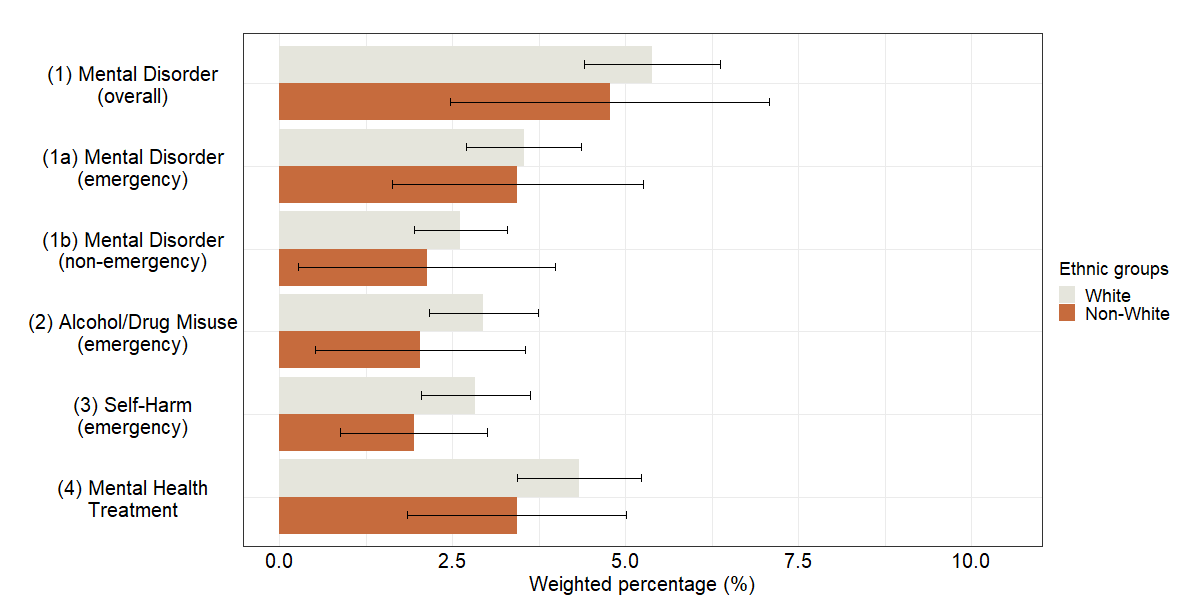


(C)


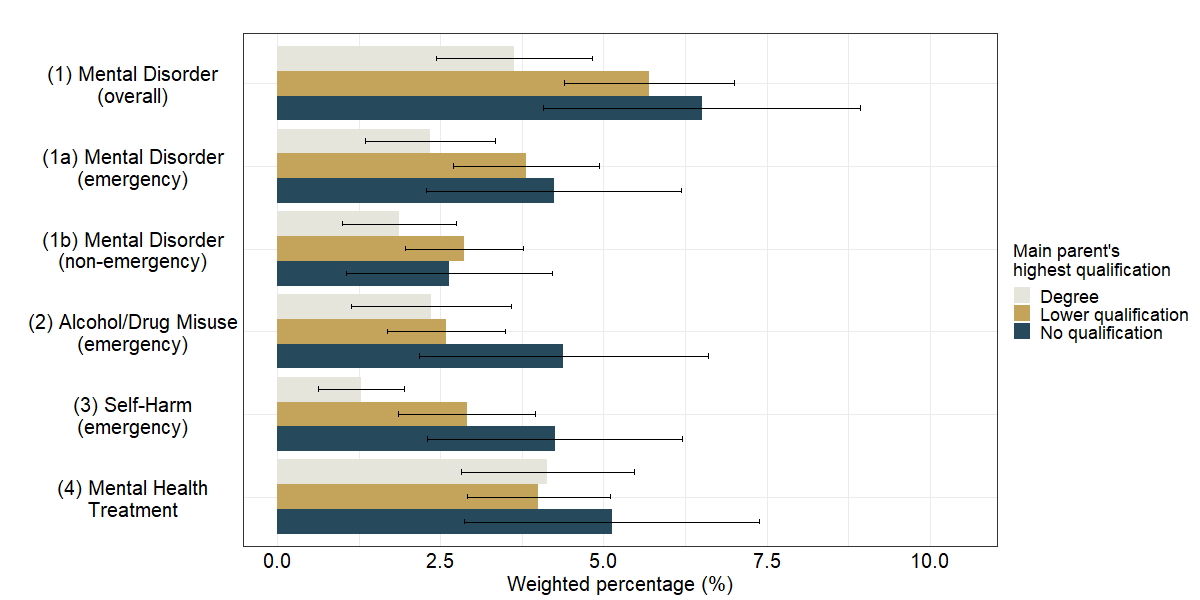


(D)


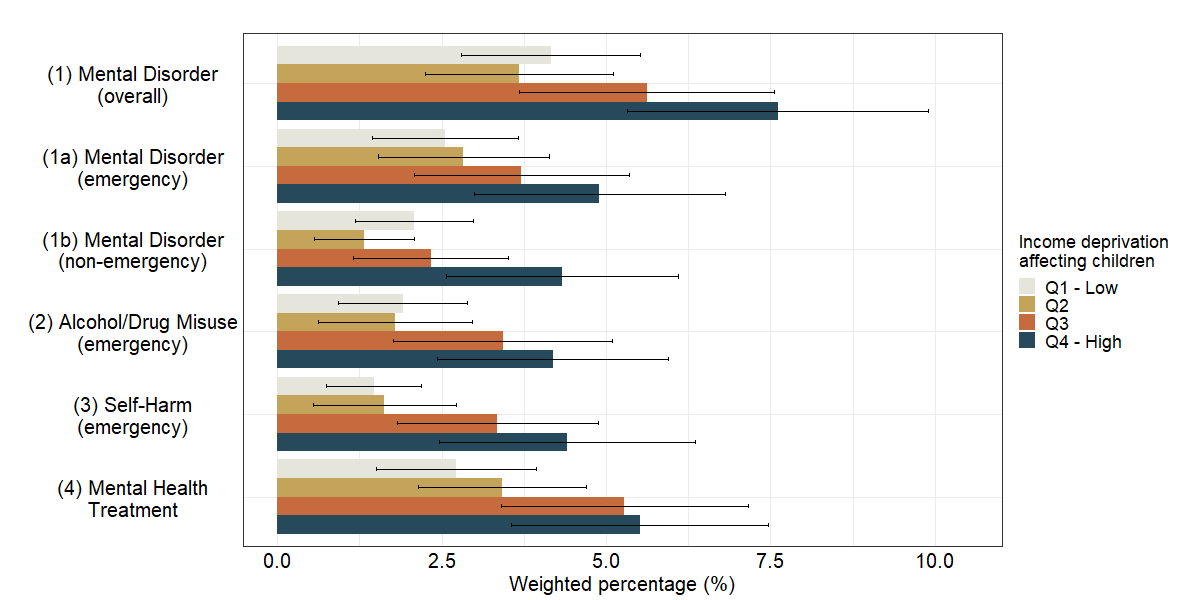


**Figure S3**: Dose-response curves between continuously measured GHQ-12 scores at age 15 and mental health-related hospital attendances from age 15 to 27 years.


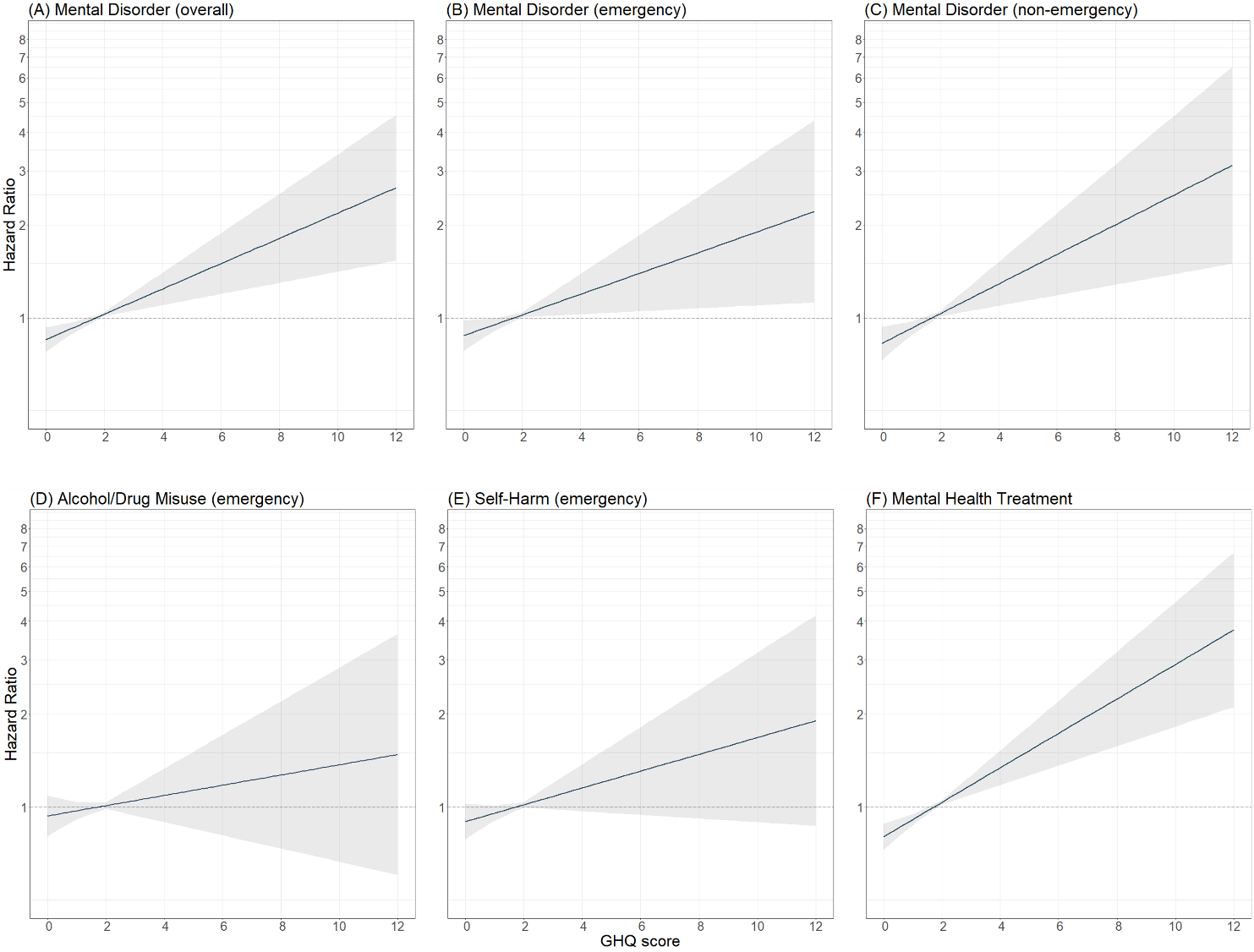


**Table S8:** Continuously measured GHQ-12 scores at age 15 and mental health-related hospital attendances from age 15 to 27 years, after multiple imputations.

|  | **Model 1** | | |  | **Model 2** | | |  | **Model 3** | | |
| --- | --- | --- | --- | --- | --- | --- | --- | --- | --- | --- | --- |
|  | HR | 95% CI | p |  | HR | 95% CI | p |  | HR | 95% CI | p |
| 1. Mental Disorder (overall) | 1.14 | 1.09-1.19 | <0.001 |  | 1.13 | 1.08-1.19 | <0.001 |  | 1.10 | 1.04-1.19 | <0.001 |
| a. Mental Disorder (emergency) | 1.13 | 1.06-1.19 | <0.001 |  | 1.12 | 1.05-1.19 | <0.001 |  | 1.08 | 1.01-1.19 | 0.02 |
| b. Mental Disorder (non-emergency) | 1.15 | 1.08-1.23 | <0.001 |  | 1.14 | 1.07-1.23 | <0.001 |  | 1.12 | 1.04-1.23 | 0.001 |
| 2. Drug/Alcohol Misuse (emergency) | 1.11 | 1.01-1.21 | 0.03 |  | 1.06 | 0.97-1.21 | 0.20 |  | 1.04 | 0.95-1.21 | 0.40 |
| 3. Self-Harm (emergency) | 1.11 | 1.04-1.19 | 0.002 |  | 1.10 | 1.03-1.19 | 0.004 |  | 1.06 | 0.99-1.19 | 0.11 |
| 4. Mental Health Treatment | 1.18 | 1.12-1.25 | <0.001 |  | 1.16 | 1.10-1.25 | <0.001 |  | 1.14 | 1.07-1.25 | <0.001 |

Cox proportionate hazard regressions were fitted with survey weights; Hazard Ratios (HR) and their 95% confidence intervals (CI) are presented. Sample size was N=4058.

Missing data was imputed by chained equations using 7 datasets. In addition to all Sweep 2 covariates used in the models, we included sex, ethnicity, ever smoked, ever drunk alcohol, income, having disability/long term illness or health problem, housing tenure, and number of parents living with the participants from Sweep 8, GHQ score from Sweep 4, Income Deprivation Affecting Children Index from Sweep 3, main parents’ living status, main parent’s highest qualification, ever smoked, ever drunk alcohol from Sweep 1, and income, and general health from Sweep 2.

Model1: adjusted for sex, age, and ethnicity.

Model2: Model 1 + main parent’s living status, main parent’s highest qualification, and Income Deprivation Affecting Children Index.

Model3: Model 2 + ever smoked, ever drunk alcohol, and having disability/long term illness or health problem.

**Table S9:** Continuously measured GHQ-12 scores at age 15 and mental health-related hospital attendance from age 15 to 27 years, after excluding participants with prior mental health hospital attendance.

|  | **Model 1** | | |  | **Model 2** | | |  | **Model 3** | | |
| --- | --- | --- | --- | --- | --- | --- | --- | --- | --- | --- | --- |
|  | HR | 95% CI | p |  | HR | 95% CI | p |  | HR | 95% CI | p |
| 1. Mental Disorder (overall) | 1.11 | 1.05-1.16 | <0.001 |  | 1.11 | 1.05-1.16 | <0.001 |  | 1.09 | 1.03-1.15 | 0.001 |
| a. Mental Disorder (emergency) | 1.09 | 1.03-1.16 | 0.005 |  | 1.09 | 1.03-1.16 | 0.005 |  | 1.07 | 1.00-1.14 | 0.06 |
| b. Mental Disorder (non-emergency) | 1.12 | 1.04-1.20 | 0.002 |  | 1.12 | 1.04-1.20 | 0.002 |  | 1.12 | 1.04-1.21 | 0.003 |
| 2. Drug/Alcohol Misuse (emergency) | 1.04 | 0.96-1.14 | 0.3 |  | 1.05 | 0.96-1.14 | 0.3 |  | 1.03 | 0.94-1.12 | 0.6 |
| 3. Self-Harm (emergency) | 1.07 | 1.00-1.15 | 0.04 |  | 1.08 | 1.01-1.16 | 0.04 |  | 1.06 | 0.97-1.14 | 0.2 |
| 4. Mental Health Treatment | 1.15 | 1.09-1.22 | <0.001 |  | 1.15 | 1.09-1.22 | <0.001 |  | 1.13 | 1.07-1.2 | <0.001 |

Cox proportionate hazard regressions were fitted using survey weights; Hazard Ratios (HR) and their 95% confidence intervals (CI) are presented. Sample size was N=4010.

Model1: adjusted for sex, age, and ethnicity.

Model2: Model 1 + main parent’s living status, main parent’s highest qualification, and Income Deprivation Affecting Children Index.

Model3: Model 2 + ever smoked, ever drunk alcohol, and having disability/long term illness or health problem.

**REFERENCES**

Blackburn, R., Ajetunmobi, O., Mc Grath-Lone, L., Hardelid, P., Shafran, R., Gilbert, R., & Wijlaars, L. (2021). Hospital admissions for stress-related presentations among school-aged adolescents during term time versus holidays in England: weekly time series and retrospective cross-sectional analysis. *BJPsych Open*, *7*(6), e215. <https://doi.org/10.1192/bjo.2021.1058>
